# Desmocollin switching in colorectal cancer

**DOI:** 10.1038/sj.bjc.6603453

**Published:** 2006-10-31

**Authors:** K Khan, R Hardy, A Haq, O Ogunbiyi, D Morton, M Chidgey

**Affiliations:** 1 Division of Medical Sciences, University of Birmingham, Clinical Research Block, Queen Elizabeth Hospital, Birmingham B15 2TH, UK; 2 Department of Surgery, Royal Free Hospital, Pond Street, London NW3 2QG, UK

**Keywords:** desmocollin, cadherin, desmosome, colorectal cancer

## Abstract

The desmocollins are members of the desmosomal cadherin family of cell–cell adhesion molecules. They are essential constituents of desmosomes, intercellular junctions that play a critical role in the maintenance of tissue integrity in epithelia and cardiac muscle. In humans, three desmocollins (Dsc1, Dsc2 and Dsc3) have been described. The desmocollins exhibit tissue-specific patterns of expression; only Dsc2 is expressed in normal colonic epithelium. We have found switching between desmocollins in sporadic colorectal adenocarcinoma with a reduction in Dsc2 protein (in 8/16 samples analysed by immunohistochemistry) being accompanied by *de novo* expression of Dsc1 (16/16) and Dsc3 (7/16). Similar results were obtained by western blotting of a further 16 samples. No change was found in Dsc2 mRNA, but *de novo* expression of Dscs 1 and 3 was accompanied by increased message levels. Loss of Dsc2 (8/19) and *de novo* expression of Dsc1 (11/19) and Dsc3 (6/19) was also found in colorectal adenocarcinomas on a background of colitis. The data raise the possibility that switching of desmocollins could play an important role in the development of colorectal cancer.

Tumour development is in part characterised by the ability of cells to overcome cell–cell adhesion and invade the surrounding tissue. Two of the principal types of intercellular junctions of epithelia are adherens junctions (AJs) and desmosomes. Adherens junctions contain classical cadherins such as the tumour suppressor gene product E-cadherin. Loss of E-cadherin and a concomitant increase in another classical cadherin, N-cadherin, is a defining feature of the epithelial to mesenchymal transition, found in embryogenesis, organ development and neoplasia (Lee *et al*, 2005). This so-called ‘cadherin switch’ has been documented in a variety of cancers (Tomita *et al*, 2000; Hardy *et al*, 2002), and is thought to confer on benign tumour cells the capacity to invade surrounding tissue and ultimately to metatasise to distant sites (Lee *et al*, 2005). The desmosomal cadherins (DCs), of which there are seven in humans, three desmocollins (Dsc1–3) and four desmogleins (Dsg1-4), are membrane-spanning adhesion molecules of desmosomes. Genetic ablation of *Dsc1* and *Dsg3* results in epidermal blistering in transgenic mice, illustrating the importance of these molecules for the maintenance of normal intercellular adhesion (Koch *et al*, 1997; Chidgey *et al*, 2001). Loss of expression of DCs has been documented in various cancers (see Chidgey, 2002; Biedermann *et al*, 2005; Oshiro *et al*, 2005) and overexpression of DCs in nonadhesive cultured fibroblasts generates adhesion and inhibits invasion (Tselepis *et al*, 1998), supporting the idea that DCs have a tumour suppressor role. In this study, we have examined the expression of the desmocollins in two groups of colorectal tumours: sporadic adenocarcinomas and colorectal adenocarcinomas arising on a background of colitis. We show for the first time that switching between nonclassical cadherins occur in neoplasia.

## MATERIALS AND METHODS

### Immunohistochemistry

Formalin-fixed, paraffin-embedded sections of sporadic colorectal adenocarcinomas and of colorectal adenocarcinomas arising on a background of colitis were obtained from the archives of University Hospital, Birmingham and the Royal Free Hospital, London respectively. Sections were examined by immunohistochemistry using the streptavidin–biotin indirect immunoperoxidase method. Antibodies JCMC against Dsc1 (North *et al*, 1996), 610120 against Dsc2 (Progen, Heidelberg, Germany) and U114 against Dsc3 (Progen) were used. Immunostained material was assessed by two independent observers and immunoreactivity compared to that of normal colon.

### Western blotting

Sporadic tumour samples with matched specimens of histologically normal, large bowel mucosa were collected with patient consent at the time of tumour resection according to local ethical guidelines. Normal mucosa was obtained from a cancer-free patient undergoing a total colectomy. Protein lysates were prepared using TRI Reagent (Molecular Research Center, Cincinnati, OH, USA) and Western blots were probed with antibodies (as above) against Dsc1, Dsc2 and Dsc3. An antibody against keratin 8 (clone C51; Zymed, San Francisco, CA, USA) was used as a loading control for epithelial protein.

### RT–PCR

Total RNA was prepared using TRI Reagent and first-strand cDNA synthesis was performed using a kit (Roche, Lewes, Sussex, UK), random primers and total RNA (1 *μ*g). For semiquantitative RT–PCR, an aliquot (1 *μ*l) of the first-strand cDNA reaction was amplified with primer pairs 1, 2 and 3 (Supplementary Table 1) against Dsc1, Dsc2 and Dsc3, respectively. Normalisation was carried out using primer pair 4 against the housekeeping gene glyceraldehyde 3′ phosphate dehydrogenase. For quantitative RT—PCR, an aliquot of the first-strand cDNA reaction was amplified with primer pairs 5, 6 and 7 (Supplementary Table 1). A Sybr Green PCR master mix (Applied Biosystems, Warrington, Cheshire, UK) was used and normalisation was carried out using primer pair 8 against the gene encoding the epithelial protein keratin 8 (Supplementary Table 1).

### Southern blotting

DNA was resolved by agarose gel electrophoresis, transferred onto Biodyne B membrane (Pall) and probed using a T4 kinase end-labelled Dsc1-specific oligonucleotide (CCAGTGGTGAAGGCTT AAGGT).

## RESULTS

### Reduced expression of Dsc2 in sporadic colorectal adenocarcinoma

**Figure 1:**
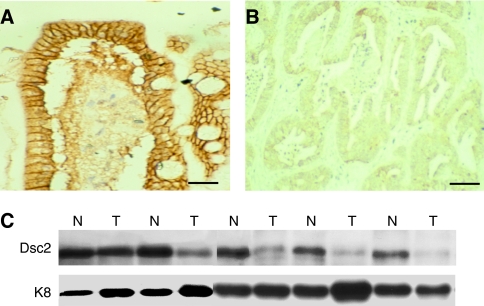
Reduced expression of Dsc2 in sporadic colorectal cancer. Dsc2 is localised at the cell membrane (brown staining) in normal colonic epithelium (**A**) but shows reduced immunoreactivity and a relocalisation from the membrane to the cell cytoplasm in colorectal adenocarcinoma (**B**). (**C**) Loss of expression of Dsc2 by Western blotting. Note that the antibody used does not detect the smaller Dsc2 ‘b’ protein. N, normal; T, tumour, K8, keratin 8 loading control for epithelial protein. Bar, 50 *μ*m.

In normal colonic epithelium Dsc2 showed strong staining at the cell membrane as expected (Figure 1A). A reduction in Dsc2 protein expression was found in eight of 16 sporadic cancer specimens examined (Figure 1B). There was no relationship between Dsc2 expression status and any of the clinicopathological variables analysed (Supplementary Table 2). In tumour samples, Dsc2 showed both a reduction in expression and a relocalisation from the membrane to the cell cytoplasm (Figure 1B). To confirm that Dsc2 expression is lost in colorectal cancer a further 16 samples were analysed by Western blotting. Reduced levels of Dsc2 protein were found in 11 out of 16 (Figure 1C).

### *De novo* expression of Dsc1 and Dsc3

**Figure 2:**
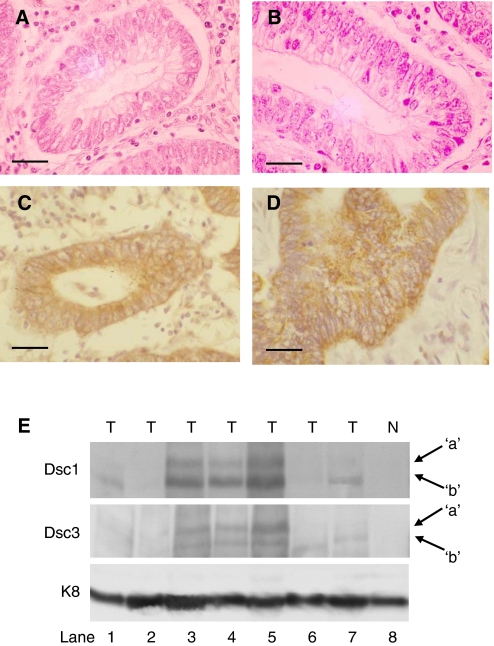
*De novo* expression of Dsc1 and Dsc3 in sporadic colorectal cancer. Dsc1 (**A**) and Dsc3 (**B**) are not expressed in normal colonic epithelial tissue, but exhibit *de novo* expression in colonic adenocarcinoma (Dsc1, **C**; Dsc3, **D**). In (**C**) and (**D**) staining is present both at the membrane and in the cell cytoplasm. (**E**) Dsc1 and Dsc3 are not present in normal colonic epithelium (lane 8), but bands of the expected size (corresponding to the Dsc ‘a’ and ‘b’ proteins) were detected by Western blotting in a subset of tumour samples. Samples in lanes 3–5 were adjudged to be positive for Dsc1 (a and b proteins) and Dsc3 (a and b), while that in lane 7 was adjudged positive for Dsc1 (a and b) alone. Bands corresponding in size to Dsc1b and Dsc3b were also detected in the samples in lanes 1 and 7 respectively. The band in lane 6 (lower panel) is nonspecific. Samples in lanes 1 and 3 showed no change in Dsc2, while those in lanes 4–7 showed a reduction in Dsc2 expression (not shown). The sample in lane 2 was not tested for Dsc2. N, normal; T, tumour. Bar, 50 *μ*m.

Dsc2 is the only desmocollin produced by simple epithelial tissues such as the colon (Nuber *et al*, 1995). To determine whether loss of Dsc2 was accompanied by *de novo* expression of Dsc1 and Dsc3, we examined sporadic colorectal cancers (those previously analysed for Dsc2 expression by immunohistochemistry) using antibodies specific for Dscs 1 and 3. As expected, no Dsc1 or Dsc3 expression was detected in normal colon (Figure 2A and B). Surprisingly, Dsc1 expression was detected in all 16 tumour samples examined (Figure 2C). In these samples, staining was found both at the cell membrane and in the cell cytoplasm. Dsc3 exhibited a similar pattern of expression in seven of 16 samples (Figure 2D). Inappropriate expression of Dsc1 and Dsc3 was restricted to morphologically abnormal glands. *De novo* expression of Dsc1 and Dsc3 was not entirely dependent on loss of Dsc2 as Dsc1 was expressed in all tumour specimens and Dsc3 was detected in three of eight samples that showed normal levels of Dsc2 (Supplementary Table 3). Loss of E-cadherin has been previously documented in colorectal cancer. We found loss of expression of E-cadherin in nine of 16 sporadic tumours. Dsc2 expression was lost in seven of nine of these, with the remaining two showing normal Dsc2 expression (Supplementary Table 3).

To confirm the presence of Dsc1 and Dsc3 in colorectal cancer, seven new samples of adenocarcinoma were compared with normal colonic epithelial tissue by western blotting. Bands of the expected molecular weight were detected in four of seven (Dsc1) and three of seven (Dsc3)(Figure 2E). No evidence was found for the presence of either DC in normal tissue (Figure 2E).

### RNA expression profile

**Figure 3:**
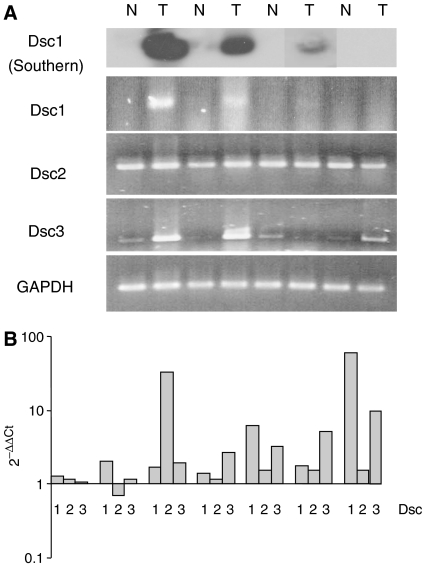
RNA encoding Dsc2 is unchanged, whereas that encoding Dsc1 and Dsc3 is increased in sporadic adenocarcinoma. (**A**) Semiquantitative RT–PCR was used to measure RNA in matched specimens of histologically normal tissue and tumour material. In each case, PCR products were of the expected size and identical in mobility to those amplified from reverse transcribed RNA from normal human epidermal keratinocytes (not shown). To confirm the presence of Dsc1 message in colorectal cancer samples, amplified DNA was blotted onto nitrocellulose and probed with a Dsc1-specific oligonucleotide (predicted to hybridise within the PCR fragment). (**B**) Levels of RNA encoding Dsc1, Dsc2 and Dsc3 were compared for a further seven matched pairs by quantitative RT–PCR.

Semiquantitative RT–PCR was used to determine whether loss of Dsc2 protein was accompanied by loss of RNA. Four pairs of matched samples, not previously examined, were used. Transcription of Dsc2 was not altered in any of the four tumours (Figure 3A). However, both Dsc1 (3/4) and Dsc3 (3/4) mRNA was clearly upregulated. To quantify changes in desmocollin expression, we examined a further seven samples by quantitative RT–PCR (Figure 3B). Similar results were found to those obtained using semiquantitative RT–PCR. Thus, although an increase in Dsc2 message levels was found in one sample, the majority (six of seven) showed no significant changes in Dsc2 transcription (i.e. there was <2-fold difference in expression levels between tumour samples and matched controls) (Figure 3B). In no case was transcription of Dsc2 downregulated. As before, increases in Dsc1 and Dsc3 mRNA (>2-fold) were detected in a significant number of cases (three of seven and four of seven tumours respectively) (Figure 3B).

A summary of the results from immunohistochemistry, Western blotting and RT–PCR is given in Supplementary Table 4.

### Desmocollin switching occurs in colorectal adenocarcinoma on a background of colitis

To determine whether desmocollin switching was exclusive to sporadic tumours, we examined expression of Dscs 1–3 in a series of colitis-associated colorectal adenocarcinomas. Analysis was restricted to immunohistochemistry as samples were available only as paraffin-embedded sections. The median age of patients with colitic tumours was less than those with sporadic tumours, but tumour stage, differentiation grade, site and microsatellite instability were comparable between groups (Supplementary Table 2). In tumours on a background of colitis, loss of Dsc2 was observed in eight of 19 specimens examined, and *de novo* expression of Dsc1 and Dsc3 was detected in 11 of 19 and six of 19 specimens respectively (Supplementary Table 4). E-cadherin expression was lost in 16 of 19 colitic tumours. Thus, in common with previous studies (Aust *et al*, 2001), we found that E-cadherin more commonly showed decreased expression in colitic rather than sporadic tumours (84 *vs* 56%). Dsc2 expression was lost in all 16 colitic tumours that showed loss of E-cadherin (data not shown).

## DISCUSSION

In this report, we show for the first time that Dsc2 protein expression is reduced in colorectal cancer, and that this is accompanied by *de novo* expression of Dsc1 and Dsc3. These changes in Dsc expression pattern may result in significant alterations in desmosome function, but do not result in the complete loss of desmosomes from colorectal cancer tissue (Collins *et al*, 1990). However, it is possible that the size and number of desmosomes is reduced (Bosch *et al*, 2005). To date, only switching between classical cadherin molecules has been described (e.g. Tomita *et al*, 2000; Hardy *et al*, 2002). The functional significance of switching between desmosomal cadherins remains unknown, but will be an important area of future investigation.

In no case was loss of Dsc2 message detected, suggesting that transcriptional mechanisms are not responsible for the loss of Dsc2 protein. The most plausible explanation for the reduction in Dsc2 protein is loss of stability. One possibility is that this could be caused by a loss of cell–cell contact, perhaps as a result of loss of expression of E-cadherin, as desmocollins are rapidly degraded in the absence of intercellular contacts (Penn *et al*, 1987). However, it should be noted that in some cases it appears that Dsc2 expression is lost in the presence of normal amounts of E-cadherin (Supplementary Table 3). No reduction in desmocollin expression was found in the only previous study of desmosomes in colorectal cancer (Collins *et al*, 1990). The most likely explanation for the discrepancy lies in the antibodies used. We have been careful to use antibodies specific for individual Dsc isoforms. Collins *et al* (1990) carried out their study before all of the desmocollin isoforms had been discovered and their antibody may have reacted with more than one isoform.

Increased amounts of message encoding both Dsc1 and Dsc3 were detected in cancer specimens. The mechanism by which this occurs is not clear. All seven DC genes are clustered in the same region of chromosome 18q (Hunt *et al*, 1999), and their expression may be linked (North *et al*, 1996; King *et al*, 1997). Induction of Dsc1 and Dsc3 RNA could be a compensatory response to the loss of Dsc2 protein. In a similar way, loss of Dsc1 protein from the epidermis of knockout mice results in large (>20-fold) increases in Dsc2 RNA (M Chidgey, unpublished). The mechanism whereby an increase in Dsc1 and Dsc3 message levels could result from a loss of Dsc2 protein (while Dsc2 mRNA remains unchanged) remains to be determined.

The changes in desmocollin expression profile were similar in both sporadic and colitis-associated colorectal cancer. These changes could significantly reduce the adhesion between colonic epithelial cells resulting in an increased propensity of the cells to proliferate, invade surrounding tissues and undergo metastasis. Switching of desmocollin isoforms could also affect the localisation of cytoplasmic desmosomal constituents, and indeed a redistribution of *γ*-catenin (plakoglobin) from the membrane to the cytoplasm does occur in both sporadic adenocarcinoma (Kolligs *et al*, 2000) and adenocarcinoma arising on a background of colitis (our unpublished data). The effects of this redistribution are unknown, but one downstream consequence of *γ*-catenin signalling is strong activation of the oncogene c-*myc* (Kolligs *et al*, 2000). It is also possible that disturbances in desmocollin expression profile could affect *β*-catenin signalling. Increased *β*-catenin transcriptional activity has been detected in both Dsc1 null mice (Merritt *et al*, submitted), and in transgenic mice that exhibit disturbances in the normal balance between desmocollin isoforms in the epidermis (Hardman *et al*, 2005). Increased *β*-catenin signalling (as a result of APC mutation) is a common causative event in colorectal cancer, and it is conceivable that DC switching could contribute to *β*-catenin dysregulation and so play a contributory role in the initiation of the early stages of colorectal tumorigenesis.
