# Supplementary material for: Desmocollin switching in colorectal cancer
Source: Br J Cancer. 2006 Oct 31;95(10):1367–70. doi: 10.1038/sj.bjc.6603453 (PMC2360607; doi:10.1038/sj.bjc.6603453)
Supplement: Supplementary Tables [file 95-6603453x1.doc]

Supplementary Table 1. Primers for PCR. GAPDH, glyceraldehyde 3’ phosphate dehydrogenase; K8, keratin 8.

Primer pair Target Sequence

1 Dsc1 TTGGATACAAAGCACTGGACC

CCAGAAAGAATTGAAAAGGTGG

2 Dsc2 AGAAGCCTGGATAGAGAGG

TCCACCGCCAATCCTTGG

3 Dsc3 GCACTCCTGCAGCCCAAT

GACACTGAGTTGGATGAGTA

4 GAPDH GCCTCCTGCACCACCAACTG

CGACGCCTGCTTCACCACCTTCT

5 Dsc1 TGCCAAACACTCCTCACTCAAA

TTCAATGGCTTGACAACACACA

6 Dsc2 CGGAGATTGTTGCGGTTGA

GGAAAGACGTGCTGCTGTATCA

7 Dsc3 GGCTGTTGCGCTGTCTGATAA

GCCCAAGGAATTCTCTTGCA

8 K8 GATCGCCACCTACAGGAAGCT

ACTCATGTTCTGCATCCCAGACT

Supplementary Table 2. Clinicopathological features of colorectal tumours analysed by immunohistochemistry. Tumours were classified by stage of local disease (T stage) and nodal status (N stage). Microsatellite instability was measured in all 16 sporadic and 10/19 colitic tumours.

Sporadic (n=16) Colitic (n=19)

Mean age of patient 68 58

Stage T1/2 5 5

T3/4 11 14

N0 9 12

N1/2 7 7

Differentiation grade Well 5 5

Moderate 7 8

Poor 4 6

Site Left 9 14

Right 6 5

Microsatellite instability ++ 2 0 (RER+ at >2 loci)

+ 3 2 (RER+ at 1 locus)

- 11 8

**Supplementary Table 3. Clinical and pathological features and pattern of desmocollin and E-cadherin expression in sporadic colorectal adenocarcinomas.** Tumours were classified by stage of local disease (T stage), nodal status (N stage) and metastatic spread (M stage). Samples were analysed by immunohistochemistry. c, cytoplasmic; m, membranous, gen, general; occ, occasional.

Tumour TNM stage Dsc1 Dsc2 Dsc3 E-cadherin

1. T3/4 N0 Mx c+m m c+m m
2. T3/4 N0 Mx c+m m absent gen loss
3. T3/4 N1/2 M1 c+m loss absent gen loss
4. T3/4 N0 Mx c+m m absent m
5. T3/4 N1/2 Mx c+m loss c+m occ loss
6. T1/2 N0 Mx c+m m c+m m
7. T3/4 N1/2 Mx c+m loss absent occ loss
8. T3/4 N1/2 M1 c+m loss c+m m
9. T3/4 N1/2 Mx c+m loss absent occ loss
10. T1/2 N0 Mx c+m loss c+m gen loss
11. T3/4 N0 Mx c+m m c+m m
12. T1/2 N0 Mx c+m loss absent occ loss
13. T3/4 N1/2 Mx c+m loss c+m occ loss
14. T1/2 N0 Mx c+m m absent occ loss
15. T1/2 N0 Mx c+m m absent m
16. T3/4 N0 Mx c+m m absent m

**Supplementary Table 4. Summary of findings.** Each technique was carried out on independent groups of samples. Figures in parentheses are percentages.

# PROTEIN

# Sporadic

Loss of Dsc2 Gain of Dsc1 Gain of Dsc3

Immunohistochemistry (n=16) 8 (50) 16 (100) 7 (44)

Western (n=17) 11/16 (69) 4/7 (57) 3/7 (43)

# Colitic

Immunohistochemistry (n=19) 8 (42) 11 (58) 6 (32)

**RNA**

**Sporadic**

No change in Dsc2 Gain of Dsc1 Gain of Dsc3

RT-PCR (n=4) 4 (100) 3 (75) 3 (75)

qRT-PCR (n=7) 6 (86) 3 (43) 4 (57)
